# Predominant Bacterial and Viral Otopathogens Identified Within the Respiratory Tract and Middle Ear of Urban Australian Children Experiencing Otitis Media Are Diversely Distributed

**DOI:** 10.3389/fcimb.2022.775535

**Published:** 2022-03-11

**Authors:** Chinh C. Ngo, Helen M. Massa, Brent A. McMonagle, Christopher F. Perry, Michael D. Nissen, Theo P. Sloots, Ruth B. Thornton, Allan W. Cripps

**Affiliations:** ^1^ John Curtin School of Medical Research, College of Health and Medicine, Australian National University, Canberra, ACT, Australia; ^2^ School of Pharmacy and Medical Sciences, Griffith University, Gold Coast, QLD, Australia; ^3^ School of Medicine and Dentistry, Griffith University, Gold Coast, QLD, Australia; ^4^ Pindara Private Hospital, Ramsay Health Care, Gold Coast, QLD, Australia; ^5^ School of Clinical Medicine, Faculty of Medicine, University of Queensland, Brisbane, QLD, Australia; ^6^ Otolaryngology, Head and Neck Surgery Department, Queensland Children's Hospital, Brisbane, QLD, Australia; ^7^ Child Health Research Centre, Faculty of Medicine, University of Queensland, Brisbane, QLD, Australia; ^8^ Centre for Child Health Research, University of Western Australia, Perth, WA, Australia; ^9^ Wesfarmers Centre of Vaccines and Infectious Disease, Telethon Kids Institute, Perth, WA, Australia

**Keywords:** otitis media, etiology, *Haemophilus influenzae*, *Streptococcus pneumoniae*, *Moraxella (Branhamella) catarrhalis*, otitis media with effusion (OME), recurrent acute otitis media (RAOM)

## Abstract

**Background:**

Otitis media (OM) is one of the most common infections in young children, arising from bacterial and/or viral infection of the middle ear. Globally, *Streptococcus pneumoniae* and non-typeable *Haemophilus influenzae* (NTHi) are the predominant bacterial otopathogens. Importantly, common upper respiratory viruses are increasingly recognized contributors to the polymicrobial pathogenesis of OM. This study aimed to identify predominant bacteria and viruses in the nasopharynx, adenoids and middle ears of peri-urban/urban South-East Queensland Australian children, with and without clinical history of chronic otitis media with effusion (COME) and/or recurrent acute otitis media (RAOM).

**Methods:**

Sixty children, 43 diagnosed with OM and 17 controls with no clinical history of OM from peri-urban/urban South-East Queensland community were recruited to the study. Respiratory tract bacterial and viral presence were examined within nasopharyngeal swabs (NPS), middle ear effusions (MEE) and adenoids, using real-time polymerase chain reaction (RT-PCR) and bacterial culture.

**Results:**

At least one otopathogen present was observed in all adenoid samples, 86.1% and 82.4% of NPS for children with and without OM, respectively, and 47.1% of the MEE from the children with OM. NTHi was the most commonly detected bacteria in both the OM and control cohorts within the adenoids (90.0% vs 93.8%), nasopharynx (67.4% vs 58.8%) respectively, and in the MEE (OM cohort 25.9%). Viruses were detected in all adenoid samples, 67.4% vs 47.1% of the NPS from the OM and control cohorts, respectively, and 37% of the MEE. Rhinovirus was the predominant virus identified in the adenoids (85.0% vs 68.8%) and nasopharynx (37.2% vs 41.2%) from the OM and control cohorts, respectively, and the MEE (19.8%).

**Conclusions:**

NTHi and rhinovirus are predominant otopathogens within the upper respiratory tract of children with and without OM from peri-urban and urban South-East Queensland, Australia. The presence of bacterial otopathogens within the middle ear is more predictive of concurrent URT infection than was observed for viruses, and the high otopathogen carriage within adenoid tissues confirms the complex polymicrobial environment in children, regardless of OM history.

## 1 Introduction

Otitis media (OM) is one of the most common infections in young children and is associated with otopathogenic bacteria and/or viruses within the upper respiratory tract (Rovers et al., 2004; Nokso-Koivisto et al., 2015; Phillips et al., 2020; Thornton et al., 2020). Globally, *Streptococcus pneumoniae*, non-typeable *Haemophilus influenzae* (NTHi) and *Moraxella catarrhalis* are the three main bacterial otopathogens of OM. *S. pneumoniae* is identified as the predominant bacterial otopathogen in the middle ear of children experiencing acute otitis media (AOM), while NTHi is more frequently detected in the middle ear of children with recurrent acute otitis media (RAOM) and/or chronic otitis media with effusion (COME) (Ngo et al., 2016). These bacteria are commensal flora in the upper respiratory tract (URT), including nasopharynx and adenoids, which are considered potential reservoirs for both bacterial and viral otopathogens causing middle ear infection (Pettigrew et al., 2012; Stol et al., 2013; Fago-Olsen et al., 2019).

Viral infection of the upper respiratory tract can contribute to OM development, through direct causation of AOM (Chonmaitree and Heikkinen, 1997; Heikkinen and Chonmaitree, 2003; Nokso-Koivisto et al., 2015; Chonmaitree et al., 2016; Schilder et al., 2016; Thornton et al., 2020) and/or initiation of inflammation prolonging middle ear effusion (MEE) (Chonmaitree and Heikkinen, 1997; Heikkinen and Chonmaitree, 2003; Nokso-Koivisto et al., 2015). A range of respiratory viruses, including adenovirus (ADV), rhinovirus (HRV) and respiratory syncytial virus (RSV), have been detected in the middle ear, nasopharynx and adenoids of children with AOM (Heikkinen et al., 1999; Chonmaitree, 2000; Heikkinen and Chonmaitree, 2003; Ishibashi et al., 2003; Monobe et al., 2003; Nokso-Koivisto et al., 2004; Ruohola et al., 2006; Bulut et al., 2007; Drago et al., 2008; Binks et al., 2011; Wiertsema et al., 2011a; Ruohola et al., 2013; Marom et al., 2019; Sawada et al., 2019). Globally, the predominant viruses identified within the middle ear, vary more widely, with RSV reported as the most frequently detected virus in the MEE of children with AOM in the United States (Heikkinen et al., 1999; Chonmaitree, 2000; Marom et al., 2019) and Asian countries (Ishibashi et al., 2003; Monobe et al., 2003; Bulut et al., 2007; Sawada et al., 2019). HRV is identified most often within the MEE of children in Australia (Wiertsema et al., 2011a) and European countries (Heikkinen and Chonmaitree, 2003; Nokso-Koivisto et al., 2004; Ruohola et al., 2006) whilst enterovirus is most frequently identified in Brazil (Buzatto et al., 2017).

Overall, otopathogen detection frequencies appear to relate to geographical location and should be actively considered. For example, in Australia, a majority of studies of OM otopathogen identification have examined rural and/or remote Australian Aboriginal children, who experience significant increased risk of severe OM. These studies include children with severe OM, chronic suppurative otitis media (CSOM) with tympanic perforation and ear discharge (Gibney et al., 2005; Leach and Morris, 2007; Smith-Vaughan et al., 2013). These studies were undertaken in the Northern Territory, a tropical region incorporating many rural and remote communities. Two studies identified bacterial and viral otopathogens within the MEE and nasopharynx of urban children with RAOM (Wiertsema et al., 2011a; Wiertsema et al., 2011b) and were undertaken in the temperate region of Western Australia. The current study aimed to identify predominant bacterial and viral carriage within the nasopharynx, adenoids and middle ears of the upper respiratory tract in peri-urban/urban children undergoing ventilation tube insertion for COME and/or RAOM in South-East Queensland, a subtropical region of Australia. The frequency of bacterial and viral co-infection and their distribution throughout the upper respiratory tracts and middle ear of children with OM were compared to the otopathogen distribution in the upper respiratory tracts of children undergoing adenoidectomy, who had no significant clinical history of OM.

## 2 Methods

### 2.1 Recruitment and Study Cohorts

Children aged 1 to 8 years old who were undergoing ventilation tube insertion (VTI) +/- adenoidectomy for the treatment of COME and/or RAOM were recruited to the OM cohort between December 2008 and December 2010 at Royal Children’s Hospital, Brisbane and January and November 2015 at Pindara Private Hospital, Gold Coast, Queensland, Australia. The clinical history of these children included: the presence of MEE for >3months or recurrent acute OM infection of either 3 episodes within 6 months or 4 or more episodes within 12 months. The cohort without OM history, recruited children of similar age undergoing adenoidectomy as treatment for adenoidal hypertrophy (AH) and/or obstructive sleep apnoea (OSA). These participants had no significant history of OM. All children recruited to this study were fully vaccinated with pneumococcal conjugate vaccines in accordance with the Australian National Immunisation Program schedule [Australian Technical Advisory Group on Immunisation (ATAGI), 2018]. Children with diagnosed immunological abnormality either intrinsic or pharmacological; anatomical or physiological defect; respiratory tract infection; purulent middle ear effusion and any malformations were excluded. All children were examined and clinically well on the day of sample collection and surgery.

This study was approved by the Children’s Health Services District Ethics Committee (2008/063 and HREC/14/QRCH/33), Greenslopes Hospital Human Research Ethics Committee (14/18) and the Griffith University Human Research Ethics Committee (MSC/05/08/HREC and MSC/19/13/HREC). Prior, informed consent was provided by each child’s parent or guardian.

A series of samples were collected from each child by the surgeon, after anesthesia induction but prior to insertion of VTI or adenoid removal. The samples collected for the OM cohort included NPS, adenoid swab/tissue and MEE, the latter were not collected from participants without OM history. All samples were placed on ice and transferred to the laboratory for processing for RT-PCR and bacterial culture within 4 hours of collection.

#### 2.1.1 Nasopharyngeal Swabs

Nasopharyngeal swabs were collected by trans-nasal insertion of a sterile flexible cotton-wool swab (Copan, Brescia, Italy) reaching the nasopharyngeal space. Swabs were stored in sterile Skim-Milk-Tryptone-Glucose-Glycerol-Broth and placed on ice until processing.

#### 2.1.2 Middle Ear Samples

Prior to MEE collection, outer ear canals were rinsed with sterile physiological saline. An anterior-inferior myringotomy incision was made and MEE from each ear was collected separately using individual sterile Argyle Specimen Traps (Covidien, Dublin, Ireland) connected to a suction system, washed through with 2ml of saline. The MEE was immediately placed on ice until processed. MEE samples were collected from both left and right ears for each OM patient.

#### 2.1.3 Adenoid Tissues

Adenoid tissue was removed using a curette and placed immediately in sterile Hanks buffered saline solution (Invitrogen, Australia) and kept on ice until transferred and processed for bacterial culture. Part of each adenoidal tissue sample was homogenized and placed in sterile Skim-Milk-Tryptone-Glucose-Glycerol-Broth for storage and RT-PCR.

### 2.2 Bacterial Culture

Following collection, MEE, NPS and adenoid samples were transported on ice to the hospital pathology laboratory and analyzed using standard pathology laboratory culture protocols (Mahon et al., 2014). All predominant bacterial colonies were recorded after 24-72h incubation and visual identification of colonies for *S. pneumoniae*, NTHi and *M. catarrhalis* were confirmed by Matrix-Assisted Laser Desorption Ionization–Time of Flight Mass Spectrometry (Shimadzu, Australia). Serotypes for *S. pneumoniae* were initially identified using a Pneumotest kit (Statens Serum Institut – (SSI), Denmark) by Quellung reaction, in accordance with manufacturer instructions. Further identification of serotypes was performed using latex agglutination (Satzke et al., 2015) with latex reagents prepared using antisera from SSI Diagnostica (Ortika et al., 2013) by the Pneumococcal Research Group at the Murdoch Children’s Research Institute (Victoria, Australia).

### 2.3 Bacterial and Viral PCR

Bacterial DNA was extracted from 300µl of MEE, NPS and adenoid samples, as previously described (Smith-Vaughan et al., 2006). RT-PCR detection of *S. pneumoniae*, NTHi and *M. catarrhalis* utilized specific primers for each bacterial target as follows: *S. pneumoniae* (autolysin gene – lytA) (Marsh et al., 2012), NTHi (haemophilus protein D gene – hpd and L-fucose permease gene – FucP) (Binks et al., 2012; Price et al., 2015) and *M. catarrhalis* (outer membrane protein gene – copB) (Marsh et al., 2012), respectively.

Viral otopathogen detection used total nucleic acid extracted using Qiagen x-tractor gene from a 200µl sample spiked with 10^4^ copies of Equine Herpes virus, before RT-PCR was performed to detect eight viral pathogens. The viruses included influenza A virus (IAV), influenza B virus (IBV), parainfluenza virus (PIV, including types 1, 2, 3), Human Adenovirus (ADV), Human metapneumovirus (hMPV), Human Respiratory Syncytial Virus (RSV), Human Rhinovirus (HRV) and WU polyomavirus (WU) by Rotorgene instruments, Qiagen Australia, as described previously (Rockett et al., 2013).

### 2.4 Statistical Analyses

Demographic data including participant age and frequency of OM episodes were compared using independent samples t-tests. Sex, URT presence of bacteria/viruses, patterns of co-colonizing or infecting species were compared between OM and control cohorts using Pearson Chi-square analyses with comparison of detection frequencies between RT-PCR and culture results performed using McNemar’s tests. For all statistical analyses, a p-value <0.05 was considered significant and all data analyses were performed using SPSS for Windows, Version 23 (IBM).

## 3 Results

### 3.1 Demographics and Sample Collection

A total of 60 children, aged 1-8 years were recruited to the OM group (mean = 3.7 years +/- 1.9 SD, n=43) and the control group without a history of OM (mean = 4.5 years +/- 1.5 SD, n=17). There were no significant differences in age or sex between the groups, as shown in **Table 1**. The number of episodes of OM differed significantly (p<0.001) between the OM group (Mean = 5.6 +/- 3.2 SD) and control group (Mean = 0.9 +/- 0.9 SD). Overall, the otopathogens identified in all sample types collected from each group are illustrated in **Supplemental Figure 1**. The otopathogens identified in the MEE samples collected and analysed for each group are shown in **Table 2**. Notably within the OM group, a MEE sample was collected from both left and right ears, however one MEE sample was not collected due to pre-existing tympanic perforation. Adenoid samples were only available for 20 children due to OM treatment decisions not including adenoidectomy. Viral RT-PCR results for two children (4 MEE samples) were excluded due to the presence of inhibitors, thus only 81 MEE samples (or 40 paired + 1 single MEE samples) from 43 recruited children were used for viral detection. In addition, one adenoid sample was not successfully collected from a control group participant (n=16) (**Table 1**).

**Table 1:** Demographic data and samples collected from peri-urban and urban children in South-East Queensland undergoing ventilation tube insertion for otitis media (OM) or adenoidectomy (Control) in the absence of a clinical history of OM.

	OM	Control
Number	43	17
Mean age in year (range)	3.7 (1-8)	4.5 (3–8)
Male (%)	23 (53.5%)	12 (70.6%)
Mean episodes of AOM within last 12 months (range)	5.6 (4-11)	0.9 (0-2)
Middle ear effusion (MEE)	85	–
Nasopharyngeal swabs (NPS)	43	17
Adenoid samples	20	16

**Table 2:** Bacterial otopathogens and viruses identified by RT-PCR in the middle ears of peri-urban and urban children in South-East Queensland undergoing ventilation tube insertion for otitis media (OM).

	OM(n=85 ears)
Bacterial otopathogens (n=85 ears)	40 (47.1%)
* S. pneumoniae*	15 (17.6%)
* H. influenzae*	22 (25.9%)
* M. catarrhalis*	17 (20.0%)
Viruses (n=81 ears)	30 (37.0%)
Adenovirus	3 (3.7%)
Human metapneumovirus	3 (3.7%)
Influenza B virus	1 (1.2%)
Respiratory syncytial virus	6 (7.4%)
Rhinovirus	16 (19.8%)
WU polyomavirus	5 (6.2%)
No pathogen	30 (37.0%)
Bacterial otopathogens alone	21 (25.9%)
Viruses alone	15 (18.5%)
Bacterial otopathogens and viruses	15 (18.5%)

Number and percentage (between brackets) of samples in which bacteria were detected. Viral detections are from 81 MEE samples due to presence of inhibitors in 4 samples and one sample not collected due to pre-existing tympanic membrane perforation.

### 3.2 Bacterial Otopathogens and Viruses Present in the Middle Ear

Within the middle ear effusate (MEE), bacterial otopathogen identification was significantly higher using RT-PCR compared to bacterial culture for each of the 3 predominant bacteria (P<0.001 for each bacterium). Bacterial otopathogens were identified in 47.1% of all MEE samples (n=85) (**Table 2**) compared to 5.9% (n=5) from bacterial culture (**Supplemental Table 1**), thus only RT-PCR data are reported further. NTHi, identified using RT-PCR, was the most common bacterium detected in the 85 MEE samples (25.9%), followed by *M. catarrhalis* (20.0%) and *S. pneumoniae* (17.6%) (**Table 2**). Overall, at least one of the three predominant bacteria were identified within either one or both ears of 26 of the 42 participants with paired MEE samples. Comparison of the left and right ear samples from the same child (n=26) showed that different bacteria were identified in each ear (76.9%, n=20 children) whilst only 6 children had the same bacteria present in each ear **(** **Supplemental Table 2**).

At least one of the viruses tested was found in 37.0% of MEE samples (n=81), with HRV (19.8%), RSV (7.4%) and WU (6.2%) detected most frequently (**Table 2**). Overall, 22 paired left and right MEE samples had virus present in one or both ears (55.0%, n=40 pairs), with 15 of these pairs having a different virus in each ear (68.2%) compared to 8 pairs showing the same virus in both the left and right ears (31.8%) (**Supplemental Table 3**).

Together these data demonstrated that 63.0% (51/81 ears) contained at least one bacteria or virus, with the remaining 30 ears (37.0%) being negative for either the three predominant bacteria causal for OM, or the selected panel of 8 common respiratory tract viruses. Bacterial detection alone (virus negative) or concurrent detection of bacteria and virus was observed in 25.9% of MEE samples, with viral detection alone observed in 18.5% of the MEE samples. With respect to concurrent bacterial and viral presence within the MEE, ADV detection is likely correlated with the presence of NTHi (r=0.321, p=0.001, **Supplemental Table 4**).

### 3.3 Bacterial Otopathogens and Viruses Present in the Nasopharynx

Bacterial identification within the nasopharynx of children with and without a clinical history of OM by RT-PCR demonstrated that at least one of the 3 predominant otopathogenic bacteria were present in 86.0% and 82.4% of NPS samples respectively. Bacteria were more commonly identified using RT-PCR than using bacterial culture (55.8% and 70.6% for OM and control cohorts respectively) thus only RT-PCR data is reported further. NTHi was identified most frequently in the NPS of children with OM (67.4%, 29/43) and the control cohort (58.8%, 10/17). Neither RT-PCR or bacterial culture data demonstrated any statistically significant difference in otopathogen identification between the OM and control groups (**Table 3** and **Supplemental Table 5**).

**Table 3:** Otopathogens and viruses identified by RT-PCR in the nasopharynx and adenoids of peri-urban and urban children in South-East Queensland undergoing ventilation tube insertion for otitis media (OM) or adenoidectomy in the absence of a clinical history of OM (Control).

Nasopharynx	OM (n=43)	Control (n=17)	*P*
Otopathogens	37 (86.1%)	14 (82.4%)	*0.718*
* S. pneumoniae*	20 (46.5%)	7 (41.2%)	*0.708*
* H. influenzae*	29 (67.4%)	10 (58.8%)	*0.528*
* M. catarrhalis*	23 (53.5%)	8 (47.1%)	*0.653*
Viruses	29 (67.4%)	8 (47.1%)	*0.143*
Adenovirus	5 (11.6%)	0 (0.0%)	*0.142*
Human metapneumovirus	3 (7.0%)	0 (0.0%)	*0.264*
Influenza A virus	1 (2.3%)	0 (0.0%)	*0.526*
Respiratory syncytial virus	2 (4.7%)	0 (0.0%)	*0.366*
Rhinovirus	16 (37.2%)	7 (41.2%)	*0.776*
Parainfluenza virus	5 (11.6%)	1 (5.9%)	*0.666*
WU polyomavirus	8 (18.6%)	0 (0.0%)	*0.056*
No otopathogen	2 (4.7%)	1 (5.9%)	*0.841*
Bacteria alone	11 (25.6%)	8 (47.1%)	*0.114*
Viruses alone	4 (9.3%)	2 (11.8%)	*0.772*
Bacteria and viruses	26 (60.5%)	6 (35.3%)	*0.091*
**Adenoids**	**OM (n=20)**	**Control (n=16)**	***P***
Otopathogens	20 (100%)	16 (100%)	
* S. pneumoniae*	13 (65.0%)	12 (75.0%)	*0.517*
* H. influenzae*	18 (90.0%)	15 (93.8%)	*0.686*
* M. catarrhalis*	13 (65.0%)	11 (68.8%)	*0.813*
Viruses	20 (100%)	16 (100%)	
Adenovirus	13 (65.0%)	7 (43.8%)	*0.202*
Rhinovirus	17 (85.0%)	11 (68.8%)	*0.244*
Parainfluenza virus	12 (60.0%)	10 (62.5%)	*0.875*
WU polyomavirus	11 (55.%)	5 (31.3%)	*0.154*
No pathogen	0 (0.0%)	0 (0.0%)	–
Bacteria alone	0 (0.0%)	0 (0.0%)	–
Viruses alone	0 (0.0%)	0 (0.0%)	–
Bacteria and viruses	20 (100.0%)	16 (100.0%)	–

Number and percentage (between brackets) of samples in which bacteria were detected. P value was analyzed by Pearson Chi-square analyses.

Viral identification showed that 67.4% and 47.1% of NPS samples contained one of the 8 viruses investigated in the OM and control groups, respectively. HRV was the most commonly detected virus in NPS samples from both cohorts, with ADV, hMPV, RSV, IAV and WU viruses detected at lower frequencies within the OM cohort only (**Table 3**).

Further examination of the frequency of co-detection of bacteria and viruses was assessed in NPS from both cohorts. Overall, the frequencies where no pathogen, and virus alone were detected within NPS were low, compared to bacteria alone, however there was no significant difference between children with and without OM (4.7% vs 5.9%, 9.3% vs 11.8% and 25.6% vs 47.1%, respectively) (**Table 3**). The frequency of co-detection of bacteria and viruses in the OM group appeared higher but did not differ significantly from the control group (60.5% vs 35.3%, p=0.091) (**Table 3**).

### 3.4 Bacterial Otopathogens and Viruses Present in the Adenoids

Adenoid sample collections were not collected from every recruited participant due to clinical treatment decisions however adenoid tissues were obtained from both the OM (n=20) and control cohorts (n=16) (**Table 1**). RT-PCR analyses confirmed concurrent bacterial and viral otopathogens within the adenoids of all children, regardless of OM history. NTHi was most frequently detected in both the OM (90.0%, n=18/20) and control cohorts (93.8%, n=15/16). There were no significant differences in bacterial identifications between OM and control cohorts (**Table 3** and **Supplemental Table 5**).

All adenoid samples, from both OM and control cohorts were positive for at least one of the 8 viruses tested. HRV was the predominant virus identified within OM (85.0%, n=17/20) and control group (68.8%, n=11/17) adenoids. ADV, PIV and WU virus were detected in adenoids from both cohorts but did not differ in frequency between the OM and control groups (**Table 3**).

### 3.5 Concurrent Bacterial Otopathogens and Viruses Present in the Same Child

**Figure 1:**
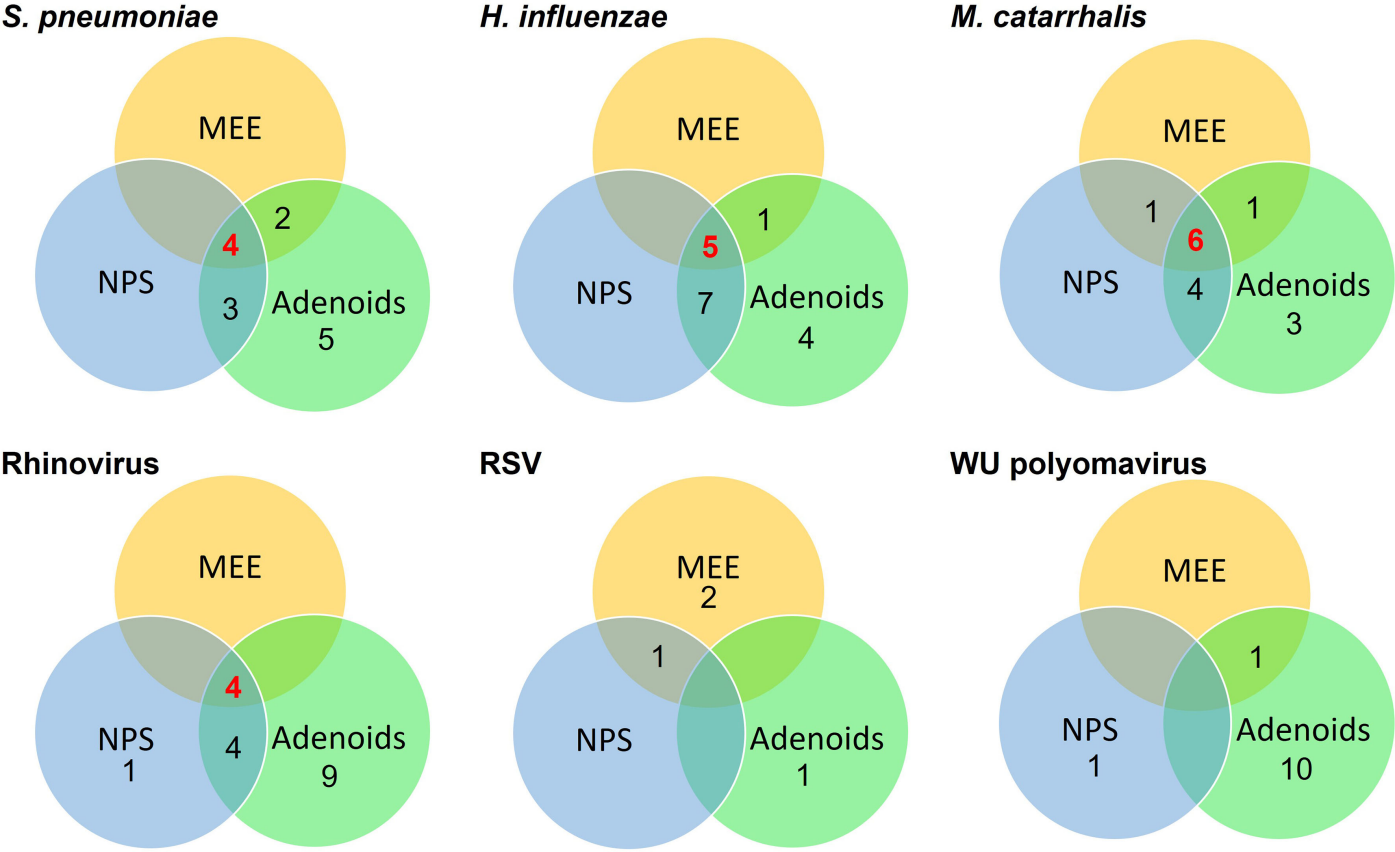
Distribution of concurrent bacterial and viral detection of predominant otopathogenic microbes within the middle ear effusate (MEE), nasopharyngeal swab (NPS) and adenoid samples of the same peri-urban/urban children in South-East Queensland who were undergoing ventilation tube insertion for otitis media (OM). Each circle represents the sample locations within each child and their intersections indicate the number of children with the same microbe identified within two or three of the sample locations.

Concurrent bacterial and viral detection of the three predominant bacteria, NTHi, *S. pneumoniae* and *M. catarrhalis* and the three most frequently detected viruses, HRV, WU polyoma virus and RSV, were examined within the upper respiratory tract of children with a history of OM, undergoing VTI for OM. For each child, the otopathogens identified within the MEE, NPS and adenoids were mapped and their concurrent presence in multiple upper respiratory tract locations within the same child are presented in **Figure 1**. These data show that when any of the 3 predominant bacteria are present within the middle ear, it is highly indicative of the same bacteria being present within the NPS and adenoids concurrently. Interestingly, only the presence of HRV within the MEE may indicate concurrent presence within the nasopharynx and adenoids, with RSV not identified as frequently in other areas of the upper respiratory tract (**Figure 1**).

### 3.6 Pneumococcal Serotypes

There were 31 pneumococcal isolates serotyped from samples collected in this study. Fourteen (45.2%) were serogroup 23 (serotype 23A was 12.9%, 23B was 32.3%), eight were serotype 11A (25.8%), four were serotype 35B (12.9%), three were serotype 16F (9.7%) and there were single isolates of serotypes 19A and 21 (3.2% each respectively).

## 4 Discussion

This study identified the predominant bacterial and viral carriage of OM in young peri-urban and urban children of South-East Queensland, Australia undergoing VTI for OM. Otopathogen presence was determined using RT-PCR in MEE, NPS and adenoid samples from the same child, who were clinically well at collection. The frequency of detection of the three predominant bacteria considered causal for OM within the URT, *S. pneumoniae*, NTHi and *M. catarrhalis*, did not differ significantly regardless of the child’s clinical history of OM. Rhinovirus was most frequently detected with the MEE of children with OM, with RSV and WU polyomavirus also observed, albeit at slightly, but not significantly lower rates of detection. Overall, regardless of the children’s history for OM, otopathogen detection was highest in the adenoids, then comparatively less in the nasopharynx and for children with OM, the middle ear. Despite significantly different geographical location and ethnicity demographics, these findings are consistent with the findings of a study of Australian Aboriginal children from Central Australia. Australian Aboriginal children are reported to experience significantly increased risk of OM and this report identified *Streptococcus* sp., *H. influenzae* and *M. catarrhalis* as common operational taxonomic units (OTUs) found in the adenoids, nasopharynx, and middle ear in these children undergoing treatment for OM (Jervis-Bardy et al., 2015). Similarly, more recent studies in Australia, Finland and Switzerland also found that these OTUs were abundant in the MEE of children with OM (Chan et al., 2016; Sillanpää et al., 2017; Brugger et al., 2019).

Interestingly, a larger study (n=143) conducted in Western Australia reported that children undergoing VTI surgery for RAOM at a younger age than our cohorts, had significantly higher rates of nasopharyngeal colonization with NTHi and *S. pneumoniae* compared to healthy controls (Wiertsema et al., 2011b). These younger children (mean age=1.7 years) were more severely affected by RAOM than observed for the older (mean age=3.7 years) children being treated for RAOM/COME in the current study. These findings perhaps reflect different OM cohorts with the more clinically recognizable, severe and repeated AOM in children undergoing VTI at an earlier age. It is recognized that for most children, OM frequency typically reduces with age, in association with facial growth (Bluestone, 1996; Swarts et al., 2013) but particularly with progressive maturation of their immune system (Wiertsema and Leach, 2009). The smaller cohort sizes in this study and older children recruited may help to explain the lack of significance between the frequencies of otopathogen detection observed between OM affected children and control children, in conjunction with physical and immunological development.

Consistent with previous publications, this study also demonstrated that RT-PCR was more sensitive than traditional culture techniques to the detection of bacterial otopathogens (Ngo et al., 2016). This is due to the organism's fastidious growth requirements and the presence of viable organisms within a biofilm, which reduce the sensitivity of detection using culture (Thornton et al., 2013; Niedzielski et al., 2021). In the current study, which was undertaken when the children were well, it is not possible to differentiate whether the magnitude of the difference between RT-PCR and culture results was technical or due to a lower bacterial load at the time of sampling. Technically, RT-PCR may detect unviable bacterial fragments, inflating detection frequencies, although previous research confirms that live bacteria present in biofilms, identified using fluorescence in situ hybridisation (FISH) are not always culturable (Thornton et al., 2013).

Co-detection of different bacteria in different locations of the URT in this study has not only confirmed the role of the nasopharynx and adenoids as potential sources of NTHi and *M. catarrhalis* but has also demonstrated that *S. pneumoniae* detection in the middle ear of children with OM is also linked to colonization of the nasopharynx and/or adenoids of the same child. In all cases where *S. pneumoniae* was isolated from the MEE, it was also isolated from either the NPS or adenoids, suggesting that *S. pneumoniae* in the middle ear has originated from the nasopharynx and/or adenoids, although further investigation, including typing studies are needed. Similarly, NTHi and *M. catarrhalis* may also have originated from the nasopharynx and/or adenoids in the current study, but not all NTHi or *M. catarrhalis* identifications within the same child were concurrently identified in the MEE, nasopharynx and/or adenoids. A report by Stol et al. (2013), has shown a genetic match between *S. pneumoniae* found in the middle ear and nasopharynx of children with RAOM/COME. Importantly, these children were undergoing VTI, similar to the current study, and were also not experiencing current active OM infection at the time of sample collection. Furthermore, only 80% of NTHi isolates from the middle ear and nasopharynx were a genetic match (Stol et al., 2013). Together, the current findings and those by Stol, suggest that NTHi or *M. catarrhalis* in the middle ears of children with OM may persist and are not necessarily seeded from those bacteria in the nasopharynx and/or adenoids.

Throughout this study, NTHi was the predominant otopathogen detected in the URT, particularly in the nasopharynx and adenoids of children without a clinical history of OM, in addition to within the middle ear of children with RAOM/COME. A limitation of this study and any other studies using a control group comprised of children undergoing surgery for adenotonsillar hypertrophy/disease, is that this pathogenic process may also impact the high prevalence of NTHi in the control group. Despite this limitation, the current results are consistent with reports from Western Australia and New Zealand (Wiertsema et al., 2011b; Mills et al., 2015; Seppanen et al., 2020) and reflect the regional prevalence of NTHi as the most common otopathogen in the middle ear and nasopharynx of children with RAOM/COME reported in our global systematic review (Ngo et al., 2016).

Importantly in this study, the predominant bacterial otopathogen, NTHi was progressively increasingly resistant to β-lactam antibiotics over the study period, rising from 14.3% (2009-2010) to 67.9% (2015) (**Supplemental Table 6**). In addition, multidrug resistance was not observed for NTHi within the 2009-2010 isolates but was present within the 2015 cohort (12/28 isolates data not shown). These findings highlight the importance of continuous surveillance for antimicrobial resistance to inform antibiotic therapy. Monitoring of serotype variants and their frequency in children of different ages undergoing a range of national immunization programs (NIP) from around the world would assist in evaluation of the potential impact of existing NIP vaccinations on OM prevalence. For example, recently, a 10-valent pneumococcal NTHi protein D conjugate vaccine was reported to reduce the frequency of middle ear infection caused by NTHi in Australian Indigenous communities (Leach et al., 2015). Development of an efficacious vaccine for NTHi has potential benefits for reduction in OM prevalence in both urban children and Indigenous Australian children, the latter children are known to experience significantly increased risk of severe OM development at a young age (Leach et al., 1994; Boswell and Nienhuys, 1996).

Limitations of the current study, in addition to those mentioned previously, include the selection of the comparative control group. Children undergoing adenoidectomy+/- tonsillectomy do not reflect a “healthy” cohort but are a sample of convenience, undergoing ear, nose and throat surgery and anesthesia, permitting collection of comparative clinical samples. Children with a clinically recognizable history of OM were excluded from control group recruitment and this is reflected in fewer OM episodes. The slightly but not significantly higher number of boys in the control group reflects recruitment is consistent with previous studies (Schupper et al., 2018) showing a higher incidence of adenoidectomy for boys. Similarly, the age range distribution between the control and OM groups is reflective of the diagnostic and clinical pathways, which tend to result in surgical resection of adenoids at a later age than for VTI due to RAOM or COME.

In the current study, despite the small sample size, *S. pneumoniae* and *M. catarrhalis* were detected at similar or lower frequencies compared to NTHi in the different regions of the URT in children with and without RAOM/COME. These results are consistent with the results of a systematic review of previous reports from the Pacific region, including Australia and New Zealand (Ngo et al., 2016).

Viral detections within the MEE of the present study occurred within 37.0% of MEE samples. The presence of these viruses, within the middle ears of OM prone children, in the absence of AOM symptomology provides support to the potential role of viruses in COME pathogenesis. Persistence of unresolving infection and presence of otopathogens in the middle ear may contribute to RAOM pathogenesis through dysregulated innate immune responses including inflammation and accumulation of middle ear effusate reflective of COME (Massa et al., 2015). Viral presence within the middle ear of OM-prone children contrasts to the sterile middle ear reported in children and adults without OM (Westerberg et al., 2009). Improved detection of viral otopathogens using RT-PCR may continue to better inform our understanding of the role of viruses within OM pathogenesis, particularly COME (Massa et al., 2015).

In this study, HRV was the predominant virus detected in all URT locations. The predominance of this virus is consistent with previous reports from Finland, the Netherlands and Western Australia, where viral detection within the middle ear was reported using PCR methods (Pitkäranta et al., 1998; Nokso-Koivisto et al., 2004; Ruohola et al., 2006; Wiertsema et al., 2011a; Stol et al., 2013). In contrast, RSV was the most common virus identified (via PCR) in the MEE of children with OM from Japan and Turkey (Monobe et al., 2003; Bulut et al., 2007). Similar studies from the US also confirmed RSV as the predominant virus within the middle ear, however these studies did not use PCR based detection methods (Heikkinen et al., 1999; Patel et al., 2007). Identification of the predominant virus within the MEE of children experiencing OM clearly varies with the region and recruitment criteria for participants in each study, including age and clinical symptomology. Furthermore, viral incidence may vary at different times within a year or between years as evidenced by a recent report from the US. The study reported significantly increased frequency of RSV detection in the URT of children with OM in the peak season of RSV, compared to the shoulder seasons of RSV (Makari et al., 2015). Therefore, the timing of patient recruitment may impact the study population detection frequencies of the otopathogen, if it occurred during the low-activity RSV seasons rather than the peak period (Chonmaitree, 2006). Each virus may present differently over the year, for example, HRV infections occur year round (Winther et al., 2006), which may increase the opportunity for detection and reporting. Regardless of which virus is predominant, an effective vaccine against that virus would benefit children with OM, who are positive for the virus, unfortunately, for the two most commonly detected viruses, HRV and RSV, vaccine development has been difficult (Campbell et al., 2015; Glanville and Johnston, 2015).

The frequency of viral detection varied by location within the URT, with the frequency of detecting at least one virus present in the NPS of children with OM tended to be higher than observed in control, although not significantly. This trend is consistent with a previous study in which children with RAOM had significantly higher frequency of viral detection in the nasopharynx compared to their control counterparts (Wiertsema et al., 2011a). In contrast, all adenoid tissues from children with or without OM in the current study had at least one virus present and this finding is consistent with previous studies whereby 90-100% adenoid samples from children with adenoidal hypertrophy, RAOM, or with OME had viruses detected (Herberhold et al., 2009; Sato et al., 2009; Szalmás et al., 2013). Interestingly, the current study showed that children with OM had higher frequencies of ADV and WU detection in both the nasopharynx and adenoids compared to children without OM. These findings are consistent with previous studies in Australia conducted on non-Indigenous children with/without OM (Wiertsema et al., 2011a) and Indigenous Australian Aboriginal children with/without the disease (Binks et al., 2011). In addition, those viruses were also detected in the middle ear of children with OM in the current study and further research is needed to investigate the potential role of these viruses in OM pathogenesis.

Finally, bacterial-viral co-infection or colonization in the URT may increase the incidence of OM development in children. Indeed, the current study showed that children with OM tended to show higher frequency of bacterial-viral detection in the nasopharynx compared to children without OM although the difference was not statistically significant (60.5% vs 35.3%). However, the role of co-occurrence of specific microbes in potentially increasing the risk of OM pathogenesis was demonstrated in the present study by the co-detection and correlation of ADV and NTHi. Further exploration of co-infection and its impact on OM pathogenesis has been provided through animal models, with elegant research using the chinchilla often considered the most representative of the human condition. Using the chinchilla model, Suzuki and Bakaletz (1994) demonstrated that intranasal inoculation with both ADV type 1 and NTHi resulted in development of more severe OM than was observed using either agent as a single pathogen (Suzuki and Bakaletz, 1994; Miyamoto and Bakaletz, 1997). In addition, intranasal inoculation of chinchillas using ADV may increase the transduction of NTHi from the nasopharynx to the middle ear and induce NTHi OM (Miyamoto and Bakaletz, 1997). Murine viral pre-infection models that utilize polymicrobial bacterial pathogens including *S. pneumoniae* and *M. catarrhalis* have demonstrated increased infection frequency and severity (Krishnamurthy et al., 2009), whilst bacterial co-infection models using NTHi and *M. catarrhalis* have also shown enhanced bacterial persistence and antimicrobial resistance (Armbruster et al., 2010). Interestingly, a study by Short et al. (2011) demonstrated that infection with influenza virus enabled development and persistence of pneumococcal OM *via* neutrophil extracellular trap (NET) induction in the mouse (Short et al., 2011). Persistent effusion, bacteria and NETs have been reported in children with RAOM (Thornton et al., 2013) and together, viral-bacterial co-infection play significant roles in the development and persistence of OM.

Overall, the current study identified NTHi and HRV as the predominant otopathogens within the URT of peri-urban and urban children, with and without COME/RAOM from South-East Queensland Australia. The presence of multiple otopathogens, both viral and bacterial within the middle ear and the upper respiratory tracts of clinically well children undergoing VTI surgery for COME/RAOM confirm the complexity of vaccine development to reduce the risk and impact of this frequent childhood disease. For the future, improved routine surveillance of otopathogens present in the URT of children experiencing OM, including affected children of differing ages from across the world, will better reflect the impact of existing vaccines and the support the development of new vaccines for OM prevention.

## Data Availability Statement

The original contributions presented in the study are included in the article/**Supplementary Material**. Further inquiries can be directed to the corresponding author.

## Ethics Statement

The studies involving human participants were reviewed and approved by the Children’s Health Services District Ethics Committee (2008/063 and HREC/14/QRCH/33), Greenslopes Hospital Human Research Ethics Committee (14/18) and the Griffith University Human Research Ethics Committee (MSC/05/08/HREC and MSC/19/13/HREC), in accordance with the National Statement on Ethical Conduct in Human Research 2007. Written informed consent to participate in this study was provided by the participants’ legal guardian/next of kin.

## Author Contributions

HM, RT and AC designed the study. HM, CP and BM developed and planned surgical collection protocols. BM, CP, HM, RT and MN designed recruitment and collection protocols and HM, BM and CP oversaw patient recruitment and sample collections. CN, TS, RT and QPID, MCRI and GCUH Pathology consortia processed samples. CN, HM, RT, TS, AC analysed data. CN and HM drafted the manuscript and all authors contributed to the article and approved the submitted manuscript.

## Funding

This research received funding support from the Royal Children’s Hospital Foundation grant (10258), Griffith Health Institute, Gold Coast Hospital Foundation Collaborative grant (40837 03/12/2729) and Financial Markets for Children Foundation grant (2008-216). CN received a Vietnam International Education Development-Griffith University scholarship.

## Conflict of Interest

The authors declare that the research was conducted in the absence of any commercial or financial relationships that could be construed as a potential conflict of interest.

## Publisher’s Note

All claims expressed in this article are solely those of the authors and do not necessarily represent those of their affiliated organizations, or those of the publisher, the editors and the reviewers. Any product that may be evaluated in this article, or claim that may be made by its manufacturer, is not guaranteed or endorsed by the publisher.
