# Supplementary material for: Predominant Bacterial and Viral Otopathogens Identified Within the Respiratory Tract and Middle Ear of Urban Australian Children Experiencing Otitis Media Are Diversely Distributed
Source: Front Cell Infect Microbiol. 2022 Mar 11;12:775535. doi: 10.3389/fcimb.2022.775535 (PMC8963760; doi:10.3389/fcimb.2022.775535)
Supplement: Supplementary file 1 [file Table_1.docx]

**Supplemental Table 1:** Otopathogens in the middle ears of peri-urban/urban children in South-East Queensland who were undergoing ventilation tube insertion for otitis media (OM), identified using bacterial culture.

|  | OM (n=85 ears) |
| --- | --- |
| *Otopathogens* | 5 (5.9%) |
| *S. pneumoniae* | 3 (3.5%) |
| *H. influenzae* | 2 (2.4%) |
| *M. catarrhalis* | 0 (0.0%) |

Number and percentage (between brackets) of samples in which bacteria were detected.
