# Supplementary material for: Predominant Bacterial and Viral Otopathogens Identified Within the Respiratory Tract and Middle Ear of Urban Australian Children Experiencing Otitis Media Are Diversely Distributed
Source: Front Cell Infect Microbiol. 2022 Mar 11;12:775535. doi: 10.3389/fcimb.2022.775535 (PMC8963760; doi:10.3389/fcimb.2022.775535)
Supplement: Supplementary file 2 [file Table_2.docx]

**Supplemental Table 2**. Bacterial identified in the left and right ears of the same child from 26 peri-urban/urban children undergoing ventilation tube insertion for otitis media in South-East Queensland.

| Patients | Ear | NTHi | *S. pneumoniae* | *M. catarrhalis* |
| --- | --- | --- | --- | --- |
| OM10002 | Left | -* | - | - |
|  | Right | **+**** | - | - |
| OM10004 | Left | - | - | **+** |
|  | Right | **+** | - | **+** |
| OM10005 | Left | **+** | - | - |
|  | Right | - | - | - |
| OM10013 | Left | - | - | - |
|  | Right | - | **+** | **+** |
| OM10015 | Left | **+** | **+** | **+** |
|  | Right | **+** | **+** | - |
| OM10018 | Left | **+** | **+** | - |
|  | Right | **+** | - | - |
| OM10020 | Left | **+** | **+** | **+** |
|  | Right | **+** | **+** | - |
| OM10022 | Left | - | **+** | - |
|  | Right | - | **+** | - |
| OM10023 | Left | **+** | - | **+** |
|  | Right | **+** | **+** | **+** |
| OM20004 | Left | - | - | - |
|  | Right | - | **+** | - |
| OM20005 | Left | - | **+** | - |
|  | Right | - | - | - |
| OM20006 | Left | - | - | - |
|  | Right | **+** | - | - |
| OM20007 | Left | - | **+** | - |
|  | Right | - | - | - |
| OM20008 | Left | - | - | **+** |
|  | Right | - | - | **+** |
| OM20010 | Left | - | - | **+** |
|  | Right | - | - | **+** |
| OM20012 | Left | - | - | - |
|  | Right | - | **+** | - |
| OM20014 | Left | - | **+** | - |
|  | Right | - | - | **+** |
| OM20015 | Left | - | - | - |
|  | Right | - | **+** | - |
| OM20016 | Left | - | - | - |
|  | Right | **+** | - | - |
| OM20023 | Left | **+** | - | **+** |
|  | Right | **+** | - | - |
| OM20024 | Left | - | - | - |
|  | Right | **+** | - | **+** |
| OM20026 | Left | **+** | - | - |
|  | Right | **+** | - | - |
| OM20028 | Left | **+** | - | - |
|  | Right | **+** | - | - |
| OM20031 | Left | **+** | - | - |
|  | Right | - | - | **+** |
| OM20035 | Left | - | - | **+** |
|  | Right | - | - | **+** |
| OM20037 | Left | **+** | - | - |
|  | Right | - | - | - |

*-: not detected by either bacterial culture or RT-PCR

**+: detected by either bacterial culture or RT-PCR or both
