# Supplementary material for: Predominant Bacterial and Viral Otopathogens Identified Within the Respiratory Tract and Middle Ear of Urban Australian Children Experiencing Otitis Media Are Diversely Distributed
Source: Front Cell Infect Microbiol. 2022 Mar 11;12:775535. doi: 10.3389/fcimb.2022.775535 (PMC8963760; doi:10.3389/fcimb.2022.775535)
Supplement: Supplementary file 3 [file Table_3.docx]

**Supplemental Table 3**: Viral identifications within the left and right ears of the same child from 22 peri-urban/urban children undergoing ventilation tube insertion surgery for otitis media in South East Queensland (2015).

| **Patient** | **Ear** | **HRV** | **IBV** | **hMPV** | **RSV** | **ADV** | **WU** |
| --- | --- | --- | --- | --- | --- | --- | --- |
| OM10002 | Left | -* | - | - | - | - | - |
|  | Right | - | - | - | - | **+**** | - |
| OM10004 | Left | **+** | - | - | - | - | - |
|  | Right | **+** | - | - | - | - | - |
| OM10005 | Left | - | - | - | **+** | - | - |
|  | Right | - | - | - | - | - | - |
| OM10008 | Left | - | - | - | - | - | **+** |
|  | Right | - | - | - | - | - | - |
| OM10012 | Left | **+** | - | - | - | - | - |
|  | Right | **+** | - | - | - | - | - |
| OM10013 | Left | - | - | - | - | - | **+** |
|  | Right | - | - | - | - | - | **+** |
| OM10014 | Left | **+** | - | - | - | - | - |
|  | Right | - | - | - | - | - | - |
| OM10015 | Left | - | - | - | - | **+** | - |
|  | Right | - | - | - | - | - | - |
| OM10016 | Left | - | - | **+** | - | - | - |
|  | Right | - | - | **+** | - | - | - |
| OM10018 | Left | **+** | - | - | **+** | - | - |
|  | Right | - | - | - | - | - | - |
| OM10019 | Left | **+** | - | - | - | - | - |
|  | Right | **+** | - | - | - | - | - |
| OM10021 | Left | **+** | - | - | - | - | - |
|  | Right | **+** | - | - | - | - | - |
| OM10023 | Left | - | - | - | - | - | - |
|  | Right | - | - | - | **+** | - | - |
| OM20006 | Left | - | - | **+** | - | - | - |
|  | Right | - | - | - | - | - | - |
| OM20007 | Left | - | - | - | - | - | - |
|  | Right | - | - | - | **+** | - | - |
| OM20008 | Left | - | - | - | **+** | - | - |
|  | Right | - | - | - | - | - | - |
| OM20012 | Left | **+** | - | - | - | - | - |
|  | Right | **+** | - | - | - | - | - |
| OM20024 | Left | **+** | - | - | - | - | - |
|  | Right | - | - | - | - | - | - |
| OM20026 | Left | - | - | - | - | - | - |
|  | Right | - | **+** | - | - | - | **+** |
| OM20031 | Left | - | - | - | **+** | - | - |
|  | Right | - | - | - | - | - | - |
| OM20035 | Left | **+** | - | - | - | - | **+** |
|  | Right | **+** | - | - | - | - | - |
| OM20037 | Left | **+** | - | - | - | **+** | - |
|  | Right | - | - | - | - | - | - |

*-: not detected by RT-PCR

**+: detected by RT-PCR

Viruses include: influenza A virus (IAV), influenza B virus (IBV), parainfluenza virus (PIV, including types 1, 2, 3), Human Adenovirus (ADV), Human metapneumovirus (hMPV), Human Respiratory Syncytial Virus (RSV), Human Rhinovirus (HRV) and WU polyomavirus (WU)
