# Supplementary material for: Predominant Bacterial and Viral Otopathogens Identified Within the Respiratory Tract and Middle Ear of Urban Australian Children Experiencing Otitis Media Are Diversely Distributed
Source: Front Cell Infect Microbiol. 2022 Mar 11;12:775535. doi: 10.3389/fcimb.2022.775535 (PMC8963760; doi:10.3389/fcimb.2022.775535)
Supplement: Supplementary file 4 [file Table_4.docx]

**Supplemental Table 4:** Co-detection of common viruses and bacterial otopathogens in the middle ears of peri-urban/urban children in South-East Queensland who were undergoing ventilation tube insertion for otitis media (OM).

|  | N | *S. pneumoniae* | NTHi | *M. catarrhalis* |
| --- | --- | --- | --- | --- |
| HRV Pos  HRV Neg  p-value | 16  65 | 2 (12.5%)  11 (16.9%)  *0.674* | 3 (18.8%)  19 (29.2%)  *0.415* | 4 (25.0%)  13 (20.0%)  *0.666* |
| RSV Pos  RSV Neg  p-value | 6  75 | 1 (16.7%)  12 (16.0%)  *0.966* | 3 (50.0%)  19 (25.3%)  *0.247* | 2 (33.3%)  15 (20.0%)  *0.476* |
| ADV Pos  ADV Neg  p-value | 3  78 | 1 (33.3%)  12 (15.3%)  *0.491* | 3 (100%)  19 (24.4%)  ***0.001*** | 0 (0%)  (21.8%)  *0.459* |
| hMPV Pos  hMPV Neg  p-value | 3  78 | 0 (0%)  13 (16.7%)  *0.521* | 1 (33.3%)  22 (28.2%)  *0.865* | 0 (0%)  17 (21.8%)  *0.459* |
| WU Pos  WU Neg  p-value | 5  76 | 1 (20.0%)  12 (15.8%)  *0.817* | 1 (20.0%)  22 (28.9%)  *0.691* | 2 (40.0%)  15 (19.8%)  *0.343* |
| Number and percentage (between brackets) of samples in which bacteria were detected. *p-value* (in bold and red when significant) as determined by Chi-Square analyses. Percentage calculated as percentage of the virus positive and virus negative value. | | | | |
