# Supplementary material for: Predominant Bacterial and Viral Otopathogens Identified Within the Respiratory Tract and Middle Ear of Urban Australian Children Experiencing Otitis Media Are Diversely Distributed
Source: Front Cell Infect Microbiol. 2022 Mar 11;12:775535. doi: 10.3389/fcimb.2022.775535 (PMC8963760; doi:10.3389/fcimb.2022.775535)
Supplement: Supplementary file 5 [file Table_5.docx]

**Supplemental Table 5:** Otopathogens identified by culture in the nasopharynx and adenoids of peri-urban/urban children in South-East Queensland who were undergoing ventilation tube insertion for otitis media (OM) or adenoidectomy in the absence of a clinical history of OM (Control).

|  | Culture | | *p* |
| --- | --- | --- | --- |
| **Nasopharynx** | OM (n=43) | Control (n=17) |  |
| Otopathogens | 24 (55.8%) | 12 (70.6%) | *0.293* |
| *S. pneumoniae* | 11 (25.6%) | 5 (29.4%) | *0.762* |
| *H. influenzae* | 14 (32.6%) | 6 (35.3%) | *0.839* |
| *M. catarrhalis* | 13 (30.2%) | 8 (47.1%) | *0.218* |
| **Adenoids** | OM (n=20) | Control (n=16) |  |
| Otopathogens | 15 (75.0%) | 12 (75.0%) | *1.000* |
| *S. pneumoniae* | 10 (50.0%) | 8 (50.0%) | *1.000* |
| *H. influenzae* | 7 (35.0%) | 5 (31.3%) | *0.813* |
| *M. catarrhalis* | 5 (25.0%) | 3 (18.8%) | *0.654* |

Number and percentage (between brackets) of samples in which bacteria were detected. P value was analysed by Pearson Chi-square analyses.
