# Supplementary material for: Predominant Bacterial and Viral Otopathogens Identified Within the Respiratory Tract and Middle Ear of Urban Australian Children Experiencing Otitis Media Are Diversely Distributed
Source: Front Cell Infect Microbiol. 2022 Mar 11;12:775535. doi: 10.3389/fcimb.2022.775535 (PMC8963760; doi:10.3389/fcimb.2022.775535)
Supplement: Supplementary file 6 [file Table_6.docx]

**Supplemental Table 6:** Percentage of beta-lactamase positive non-typeable *Haemophilus influenzae* (NTHi) isolated in peri-urban and urban children in South-East Queensland undergoing ventilation tube insertion for otitis media or adenoidectomy for obstructive sleep apnoea in the absence of a clinical history of OM, between 2009 and 2015.

|  | Negative for beta-lactamase (%) | Positive for beta-lactamase (%) |
| --- | --- | --- |
| NTHi samples isolated from all sites in all children (2009-2010) (n=7) | 85.7% | 14.3% |
| NTHi samples isolated from all sites in all children (2015)  (n=28) | 32.1% | 67.9%^*^ |

^*^Significantly more isolates positive for beta-lactamase in 2015 (Pearson Chi-square) p=0.042
