# Supplementary material for: Predominant Bacterial and Viral Otopathogens Identified Within the Respiratory Tract and Middle Ear of Urban Australian Children Experiencing Otitis Media Are Diversely Distributed
Source: Front Cell Infect Microbiol. 2022 Mar 11;12:775535. doi: 10.3389/fcimb.2022.775535 (PMC8963760; doi:10.3389/fcimb.2022.775535)
Supplement: Supplementary file 7 [file Image_1.pdf]

Supplemental Figure 1

This figure illustrates a summary of the concurrent bacterial and viral otopathogens detected within the middle ear effusion, nasopharynx and adenoid samples of peri-urban/urban children in South-East Queensland who were undergoing either ventilation tube insertion for otitis media (COME/RAOM) or adenoidectomy +/- tonsillectomy as treatment for adenoidal hypertrophy and/or obstructive sleep apnoea (Control). Middle ear effusion samples were not collected from control patients.

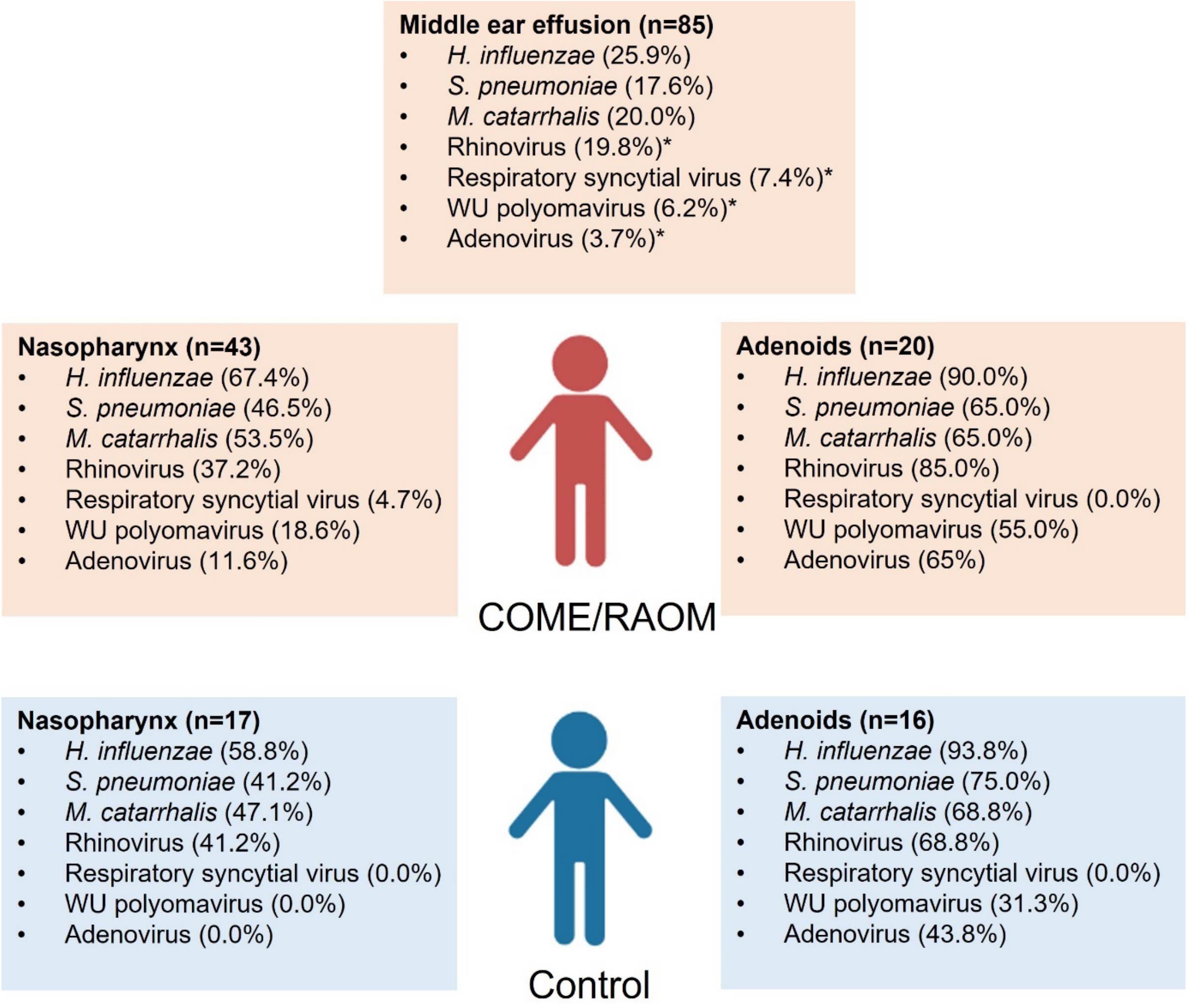

* Viral detection from 81 middle ear effusion samples
